# Resveratrol reduces inflammatory response and detrimental effects in chronic cerebral hypoperfusion by down-regulating stimulator of interferon genes/TANK-binding kinase 1/interferon regulatory factor 3 signaling

**DOI:** 10.3389/fnagi.2022.868484

**Published:** 2022-07-22

**Authors:** Ning Kang, Yuanyuan Shi, Jiaxi Song, Fei Gao, Mingyue Fan, Wei Jin, Yaran Gao, Peiyuan Lv

**Affiliations:** ^1^Department of Neurology, Hebei Medical University, Shijiazhuang, China; ^2^Department of Neurology, Hebei General Hospital, Shijiazhuang, China; ^3^Department of Oncology, Hebei General Hospital, Shijiazhuang, China

**Keywords:** chronic cerebral hypoperfusion, neuroinflammation, STING/TBK1/IRF3 pathway, resveratrol, white matter injury, microglia, neutrophil

## Abstract

Inflammatory responses induced by chronic cerebral hypoperfusion (CCH) play a critical role in the progression of vascular dementia. Stimulator of interferon genes (STING) signaling function as a key mediator of inflammation and immunological responses in the central nervous system (CNS), and resveratrol (RES) exerts potent anti-inflammatory effects. However, the role of STING signaling and the relationship between RES and STING signaling in persistent hypoperfusion-induced cerebral inflammation remain unclear. In this study, Sprague–Dawley rats were subjected to either Sham or bilateral common carotid artery occlusion (2VO) surgery and received RES or vehicle daily by intraperitoneal injection for 4 or 8 weeks. Morris’s water maze was used for the analysis of cognitive function. The neuroinflammatory responses in white matter and hippocampus of the rat brain were assessed by Western blot, Immunofluorescence staining, and qRT-PCR analyses. Myelin integrity, neutrophil infiltration, and microglia proliferation were assessed by Immunohistochemistry and histologic analysis. We demonstrated that after CCH, neurons, microglia, and astrocyte under endoplasmic reticulum (ER) stress upregulated the expression of STING, TANK-binding kinase 1 (TBK1), and the transcription factor interferon regulatory factor 3 (IRF3), as well as translocation of IRF3 into the nucleus. These were accompanied by infiltration of neutrophils, activation of microglia, and overproduction of proinflammatory mediators. Improvements in cognitive deficits were related to reduced hippocampal neuronal cell death and increased myelin integrity in RES-treated rats. The neuroprotective effects of RES were associated with suppression of the expression of tumor necrosis factor-alpha (TNF-α), intercellular adhesion molecule 1 (ICAM-1), VCAM-1, interferon-β (IFN-β), and IL-1β, likely through mitigation of the STING/TBK1/IRF3 pathway. These inhibitory effects exerted by RES also inhibited the levels of myeloperoxidase, reduced excess expression of reactive astrocytes, and activated microglia. In conclusion, the STING/TBK1/IRF3 axis may be critical for proinflammatory responses in cerebral tissue with persistent hypoperfusion, and RES exerts its anti-inflammatory effects by suppressing STING/TBK1/IRF3 signaling.

## Introduction

Vascular dementia (VaD), which presents with cognitive deficits and executive dysfunction, is the second most common cause of dementia. VaD causes cognitive impairment, induces disconnect from the outworld, burdens a patient’s family, and remains a major challenge to worldwide public health (O’Brien and Thomas, 2015). The sustained inflammation that occurs during cerebral hypoperfusion is critical pathophysiology of VaD (Lecordier et al., 2021). In chronic cerebral hypoperfusion (CCH), neuroinflammation, which is characterized by the activation of microglia and astrocytes, contributes to neuronal loss and white matter lesions, and these effects lead to learning and memory dysfunction (Hase et al., 2018).

Stimulator of interferon genes (STING/MITA; encoded by TMEM173), predominantly resides in the endoplasmic reticulum and is able to confer broad viral resistance (Liu et al., 2015). Specifically, activation of STING recruits TANK-binding kinase 1 (TBK1), subsequently, TBK1 phosphorylates STING and the transcription factor interferon regulatory factor 3 (IRF3), then IRF3 translocates into the nucleus to potentiate type I Interferons (IFN-I) responses (Liu et al., 2015). Previous studies have demonstrated that the STING/TBK1/IRF3 signaling pathway can be activated by endoplasmic reticulum (ER) stress (Ishikawa and Barber, 2008) in alcoholic liver disease (Petrasek et al., 2013) and traumatic brain injury (TBI) (Barrett et al., 2020).

Sustained STING stimulation may play a key role in inflammatory diseases. The STING-regulated inflammatory response contributes to pressure overload-induced cardiac hypertrophy (Zhang Y. et al., 2020). Obesity promotes mtDNA release into the cytosol, where it triggers activation of the STING/TBK1/IRF3 pathway and chronic inflammatory responses in adipose tissue (Bai et al., 2017). In a mice model of sepsis-induced cardiomyopathy, the activation of STING/IRF3 leads to inflammatory reactions and further increases the expression of the NLRP3 inflammasome, while STING knockdown suppresses myocardial and serum inflammatory cytokines and alleviates cardiac function (Li et al., 2019). Thus, a growing body of evidence suggests that STING is a critical signaling molecule in inflammation (Decout et al., 2021).

Increasing studies have provided evidence of the regulatory role of STING/TBK1/IRF3 signaling in various neurological diseases (Paul et al., 2021). In a rat model of ataxia-telangiectasia, unrepaired damage to DNA leads to significant levels of cytosolic DNA in Atm-deficient neurons and microglia, activates the STING pathway, increases inflammatory microenvironment, and results in neuronal dysfunction and death (Quek et al., 2017). Both genomic and mitochondrial DNA can trigger STING signaling and drive neurodegeneration in the substantia nigra pars compacta, which leads to Parkinson’s disease progression (Sliter et al., 2018). In an experimental model of stroke, cerebral ischemia promotes the release of self-derived dsDNA into the cytosol, which activates STING *via* cyclic GMP–AMP synthase (cGAS) and triggers inflammatory responses (Li Q. et al., 2020). The expression of STING mRNA is upregulated early and persistently until 60 days after TBI and is associated with chronic neurological deficits, lesions, and hippocampal neurodegeneration (Barrett et al., 2020). Although STING exhibits important anti-inflammatory effects in various CNS disease models, the role of STING in VaD has not been evaluated. Here, we sought to determine whether STING, TBK1, and IRF3 are involved in CCH injury.

Pharmacological alleviation of the inflammatory response is one of the most promising avenues for VaD therapy. Resveratrol (RES) exhibits pleiotropic actions, including the induction of neuroprotection during cognitive decline through anti-inflammatory and antioxidative activity, activation of autophagy, and inhibition of neuronal apoptosis (Griñán-Ferré et al., 2021). The suppression of inflammatory factor TBK1, which mediates the transcriptional activation of NF-κB, AP-1, and IRF3, contributes to the broad-spectrum inhibitory activity of RES (Kim et al., 2011). Additionally, RES can improve neuroimmune dysregulation by inhibiting the kinase activity of TBK1 and the activation of IRF3 *in vitro* (Youn et al., 2005). However, whether RES can protect a brain with persistent hypoperfusion by regulating TBK1/IRF3 remains unknown.

Given the well-described role of the STING/IRF3 pathway in inflammatory and neurological diseases, we used a rat model of 2VO to assess the hypotheses that STING/TBK1/IRF3 signaling exerts deleterious effects on cerebral hypoperfusion and that RES might suppress the inflammation induced by CCH through dampening the STING/TBK1/IRF3-mediated pathway.

## Materials and methods

### Experimental animals

Adult male Sprague–Dawley rats (aged 8–10 weeks and weighing 240–260 g at the beginning of the study) were purchased from Vital River Laboratory Animal Technology Co. Ltd, Beijing, China. Animals were housed in a humidity-controlled room on a 12-h light/dark cycle at 22°C ± 3°C, with free access to food and water.

### Animal surgery

Bilateral common carotid artery occlusion surgery was performed as previously described (Farkas et al., 2007). Briefly, rats were anesthetized intraperitoneally with 2% sodium pentobarbital (3 mg/kg). The right and left common carotid artery was doubly ligated with 4–0 silk sutures and cut between the ligations. Rats in the sham group were operated on without ligation.

### Experimental groups and treatments

A total of 96 rats were randomly divided into six groups as follows: (1) Sham 4w group (animals received sham operation and an equal volume of vehicle for 4 weeks); (2) Sham 8w group (animals received sham operation and an equal volume of vehicle for 8 weeks); (3) 2VO 4w group (animals underwent 2VO and treated with vehicle for 4 weeks after surgery); (4) 2VO 8w group (vehicle-treated, sacrificed at 8 weeks after 2VO); (5) 2VO + RES 4w group (RES-treated, sacrificed at 4 weeks after 2VO); (6) 2VO + RES 8w group (RES-treated, sacrificed at 8 weeks after 2VO).

**FIGURE 1:**
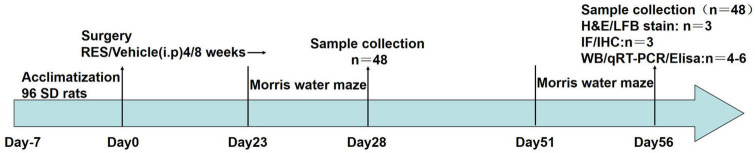
A complete flow chart of this study.

Resveratrol (R8350, Solarbio, Beijing, China) was dissolved in 0.05% DMSO prepared with 0.9% NaCl to a final concentration of 20 mg/ml. RES-treated rats received daily intraperitoneal injections of RES solution (dosage: 20 mg/kg/day) for 4 or 8 weeks after 2VO. RES doses were selected based on previous studies (Anastácio et al., 2014; Gocmez et al., 2019). The timeframe of this study is presented in Figure 1.

In another separate experiment, STING-specific palmitoylation inhibitor H-151 (HY-112693, CAS No.: 941987-60-6, MedChemExpress, United States) was used. Rats were randomly divided into four groups (*n* = 8): Sham-operated (Sham group), 2VO model (2VO group), a 2VO-operated rat with H-151 administration from day 22 to day 28 (2VO + H-151 group), and a Sham-operated rat with H-151 administration from day 22 to day 28 (Sham + H-151 group). H-151 treated rats were injected intraperitoneally daily with 500 nmol H-151 per rat in 200 μl PBS 0.1% Tween-80 from day 22 after 2VO or sham operation for successive seven days. The dose of H-151 was determined according to a previous study (Haag et al., 2018).

### Morris water maze task

Learning and spatial memory deficiencies caused by cerebral chronic hypoperfusion in rats were evaluated with the Morris Water Maze (Shanghai Jiliang Software Technology Company, Shanghai, China). Navigation trials were conducted for 5 days at the same time each day. Each rat was placed into a water-filled tank with a platform from four different quadrants. Escape latency was the time required for a rat to find and stay on the platform for more than 5 s. If a rat failed to find the platform within 120 s, it was guided to the platform and allowed to stay there for 20 s, the latency would be recorded as 120 s. For spatial probe trials, on the 6th day, the platform was removed, and the rats were placed in water at the quadrant farthest from the original platform location. The swimming route in 120 s was recorded. Spatial learning and memory functions were analyzed from the escape latency, the number of platform crossings, and the amount of time spent in the target quadrant.

### Hematoxylin–eosin staining

Brain tissues were fixed in 4% paraformaldehyde for 24 h. Coronal sections of the hippocampus CA1 area were cut at 5-μm thick. The slides were deparafinized and then stained with hematoxylin and eosin. The sections were examined by a light microscope (Nikon, NI-SS, 933313).

### Western blotting

Exactly 4 or 8 weeks after the construction of 2VO, total proteins were extracted from the white matter and hippocampus utilizing ice-cold RIPA Lysis Buffer (R0010, Solarbio), PMSF (P0100, Solarbio), Protease inhibitor cocktail (P8340, Sigma, United States), Protein phosphatase inhibitors mix (P1260, Applygen technologies, Beijing, China). Nuclear and cytosolic protein for IRF3 were prepared with Nuclear-Cytosol Extraction Kit (P1200, Applygen). A BCA protein assay reagent kit (PC0020, Solarbio) was utilized to determine protein concentrations. Equal protein sample (50 μg total protein samples, 30 μg nuclear and cytosolic protein samples) was subjected to sodium dodecyl sulfate-polyacrylamide gel electrophoresis (SDS-PAGE). Proteins were then transferred to polyvinylidene difluoride (PVDF) membranes (Millipore, Billerica, MA, United States) using a semi-dry transfer apparatus (BioRad) at 4°C with a steady current. Membranes were blocked with 5% skimmed milk in TBST (TBS and 0.1% Tween-20) for 1 h at room temperature and incubated with primary antibodies in 5% skimmed milk in TBST at 4°C overnight. Various primary antibodies were used, including Rabbit anti-TBK1 antibody (#ab40676, 1:4,000, Abcam), anti-myelin basic protein antibody (MBP, #ab40390, 1:1,000, Abcam). Phospho-PERK (PKR-like ER kinase; Thr980) (16F8) Rabbit mAb (#3179, 1:1,000), phospho-TBK1(Ser172) (D52C2) rabbit mAb (#5483, 1:1,000), and phospho-IRF-3 (Ser396) (D6O1M) rabbit mAb (#29047, 1:1,000) were purchased from Cell Signaling Technology (Beverly, MA, United States). IRF3 rabbit antibody (#11312-1-AP, 1:1,000) and TMEM173/STING rabbit antibody (#19851-1-AP, 1:1,500) were purchased from Proteintech Biotechnology (Wuhan, China). Myeloperoxidase (MPO) light chain(C-3) mouse mAb (#sc-390109, 1:200) and PERK (B-5) mouse mAb (#sc-377400, 1:200) were purchased from Santa Cruz Biotechnology (Dallas, TX, United States). The following day, membranes were rinsed three times for 10 min with TBST prior to being incubated with HRP-conjugated secondary antibodies (goat anti-rabbit, BS13278, 1:10,000 or goat anti-mouse, BS12478, 1:8,000; Bioworld, diluted in TBST) for 1h at room temperature. Again, membranes were washed with TBST thrice. The relative density of every band was detected using an ECL Western Blot Kit (CW0049S, Cwbio, China) and determined with the imaging machine (Minichemi 610 Plus, Sagecreation, Beijing, China). The relative density of every band was quantified with ImageJ software (NIH, United States). Histone-H3 (#17168-1-AP, 1:2,000, Proteintech) and β-actin (#AP0060, Rabbit, 1:10,000, Bioworld) were used as loading control.

### Quantitative real-time PCR

Brain tissues were collected from the white matter. Total RNA was extracted by TRIzol (#DP424, Tiangen Biotech, Beijing, China) following the manufacturer’s instructions, and quantified with a NanoDrop 2000 spectrophotometer (Thermo Fisher Scientific, Waltham, MA, United States). The absorbance of RNA was determined at a wavelength of 260 and 280 nm with a UV spectrophotometer. Reverse transcription was performed using a SureScript TM First-Strand cDNA Synthesis Kit (GeneCopoeia, Guangzhou, China) for quantitative PCR (ABI 7500 real-time PCR System, Applied Biosystems, CA, United States) using a fluorescent dye (BlazeTaqtm SYBR^@^ Green qPCR Mix; GeneCopoeia). RNA quantities of target genes were calculated by the 2^–ΔΔCt^ method. The final results were expressed as RQ values. The primer sequences were as follows:

IFN-β (forward, 5′- TTGCGTTCCTGCTGTGCTTCTC-3′;

reverse, 5′-TCCGTCCTGTAGCTGAGGTTGAG-3′);

ICAM-1(forward, 5′-TGTCGGTGCTCAGGTATCC ATCC-3′;

reverse, 5′-GTCTTTCATCCAGTTAGTCTCCAACCC-3′);

VCAM-1(forward, 5′-AAGTGGAGGTCTACTCAT TCCC-3′;

reverse, 5′- GGTCAAAGGGGTACACATTAG-3′);

TNF-α (forward, 5′- ATGGGCTCCCTCTCATCAG TTCC-3′;

reverse, 5′-CCTCCGCTTGGTGGTTTGCTAC-3′);

IL-1β (forward, 5′-AATCTCACAGCAGCATCTCGA CAAG-3′;

reverse, 5′-TCCACGGGCAAGACATAGGTAGC-3′);

IL-10 (forward, 5′-GGCAGTGGAGCAGGTGAAG AATG-3′;

reverse, 5′-TGTCACGTAGGCTTCTATGCAGTTG-3′);

CXCL10 (forward, 5′-TCCTGTCCGCATGTTGAGATC ATTG-3′;

reverse, 5′-ACCTTCTTTGGCTCACCGCTTTC-3′);

CD16 (forward, 5′-GACGCAACAACATATCTTCAGC ATCC-3′;

reverse, 5′-GAGTCCTATCAGCAGGCAGAAAGTG-3′);

CD206 (forward, 5′-GACAGACGGACGAGGAGTTCAT TATAC-3′;

reverse, 5′-CCACCAATCACAACAACACAGTCAAC-3′);

MBP (forward, 5′-TCTGGAAAGCGAGAATTAGCAT CTGAG-3′;

reverse, 5′-ACTGTCTTCTGAGGCGGTCTGAG-3′);

STING (forward, 5′-CAGCCTGATGAGCCTTTGG ATGAC-3′;

reverse, 5′-GGACTGGACATGGCACAACTCTTC-3′);

TBK1 (forward, 5′-GTGCTAAGGAAGGACCATCAGAA GAAG-3′;

reverse, 5′-GGCTGCGTGGTAGAATGTGACTC-3′);

IRF3 (forward, 5′-CTTACGACAGGACGCACAG ATGG-3′;

reverse, 5′-CAGGTTGACAGGTCTGGCTTATCC-3′);

GAPDH (forward, 5′-TGACGTGCCGCCTGGAGAAA-3′;

reverse, 5′-AGTGTAGCCCAAGATGCCCTTCAG-3′).

All reactions were run in triplicate. The reaction conditions were as follows: 95°C for 15 min, followed by 40 cycles of 95°C for 10 s and 60°C for 32 s.

### Immunofluorescence double staining

The localization of PERK, STING, p-TBK1, and p-IRF3 were identified by immunofluorescence. Paraffin sections of brain tissue were dewaxed with xylene and dehydrated with gradient alcohol. Antigen repair was performed with EDTA (pH 8) and heated in the microwave. After cooling naturally, the sections were washed thrice with phosphate-buffered saline (PBS, pH 7.4) and blocked using 5% bovine serum albumin (BSA; #BS114, Biosharp) for 1 h at room temperature. Subsequently, slices were incubated overnight at 4°C with mouse anti-GFAP (glial fibrillary acidic protein, #GB12096,1:500, Servicebio) to identify astrocytes, mouse anti-ionized calcium-binding adaptor molecule 1 (Iba-1; #GB12105,1:500, Servicebio) to identify microglia, mouse anti-NeuN (Neuronal Nuclei, #K009907M,1:200, solarbio) and primary antibody against PERK (1:50), STING (1:100), p-TBK1(1:100), and p-IRF3 (1:200), respectively. After three washes with PBS on the next day, slices were incubated in Cy3-conjugated anti-rabbit (GB21303,1:200, Servicebio) and AlexaFluor488-conjugated anti-mouse (GB25301,1:400, Servicebio) secondary antibodies for 50 min in the dark. Finally, DAPI (G1012, Servicebio) was added to the sections for 15 min to identify the nuclei. Images were acquired on an inverted fluorescence microscope (Eclipse C1; Nikon) at 40× objective and an imaging system (DS-U3; Nikon).

### Immunohistochemical staining

Immunohistochemical staining of coronal brain slices was performed. Briefly, deeply anesthetized rats were perfused intracardially with cold PBS and followed by 4% paraformaldehyde (PFA). Brain tissues were fixed in 4% PFA in PBS (0.01 M, pH 7.4) over 24 h at 4°C and dehydrated in gradient alcohol, and embedded in paraffin. Coronal sections were cut at 5-μm thickness using a Leica^®^ RM1850 rotary microtome (Leica Microsystem, IL, Hesja, Germany). Antigen retrieval was performed by heating in citric acid buffer (pH 6) in the microwave, followed by three washes with PBS (pH 7.4). The sections were then incubated with 3% H_2_O_2_ to eliminate the endogenous peroxidase activity for 25 min, washed three times with PBS, blocked with 3% BSA for 30 min, and incubated overnight at 4°C with anti-Iba-1 (1:100), anti-MBP (1:100) and anti-MPO (1:100) primary antibodies. The next day, the sections were rinsed with PBS and incubated with horseradish peroxidase (HRP)-labeled anti-rabbit (GB23303,1:200, Servicebio) and anti-mouse (GB23301,1:200, Servicebio) secondary antibodies at 37°C for 40 min. Slices were developed with diaminobenzidine and counterstained with hematoxylin. The samples were then dehydrated in a graded series of alcohol, cleared in xylene, and examined under a light microscope (Nikon, NI-SS, 933313, Tokyo, Japan). Cell number and positive area were quantified by ImageJ software (NIH, United States).

### Luxol fast flue staining

Luxol fast blue (LFB) staining was performed as previously described (Li M. et al., 2020). Rats were deeply anesthetized, and transcardially perfused with PBS followed by 4% PFA. Brain sections were fixed in 4% PFA for 24 h. Coronal sections containing corpus callosum were embedded in paraffin and cut at 5-μm thick that were deparafinized in xylene, absolute ethanol, and 75% ethanol. Then, the slices were incubated in LFB solution (G1030, Servicebio) for 4 h at 60°C. The slices were placed in a 0.05% lithium carbonate solution followed by 70% ethanol. To acquire images, light microscopy (Nikon, NI-SS, 933313) was used. The positive area was quantified by ImageJ software.

### Myeloperoxidase activity and 2′3′-cGAMP level assay

Samples were rinsed, weighed, and then homogenized using freezing Dounce tissue grinder (JXFSTPRP-CL, XinJing, Shanghai, China). The MPO assay kit (A044-1-1, Jiancheng Bio, Nanjing, China) was used for MPO activity measurement according to the manufacturer’s instructions. The levels of 2′3′-cGAMP, which is naturally synthesized by activated cGAS, were quantified by ELISA kit (501700, Cayman Chemical, United States) according to the manufacturer’s instructions. Absorbance at 450 nm was measured using a microplate reader (Multiskan FC, Thermo Fisher) and normalized to overall protein content. MPO activity of white matter was expressed as U/g tissue. The 2′3′-cGAMP levels in the white matter and hippocampus homogenates were expressed as pg/mg protein.

### Statistical analysis

Quantitative data were expressed as mean ± *SD*. Statistical analysis was performed by SPSS version 19 software (IBM, United States). Escape latency and frequency in the platform quadrant from the Morris Water Maze test were analyzed by repeated-measures ANOVA, with Tukey *post hoc* test (if equal variances were assumed) or Tamhane’s T2 test (if equal variances were not assumed). One-way ANOVA followed by SNK and LSD tests were used for comparison between groups. The difference was considered significant when *P* < 0.05. Graphs were drawn by GraphPad Prism 6 software (GraphPad Software, La Jolla, CA, United States).

## Results

### Resveratrol improves cognitive function recovery after chronic cerebral hypoperfusion

**FIGURE 2:**
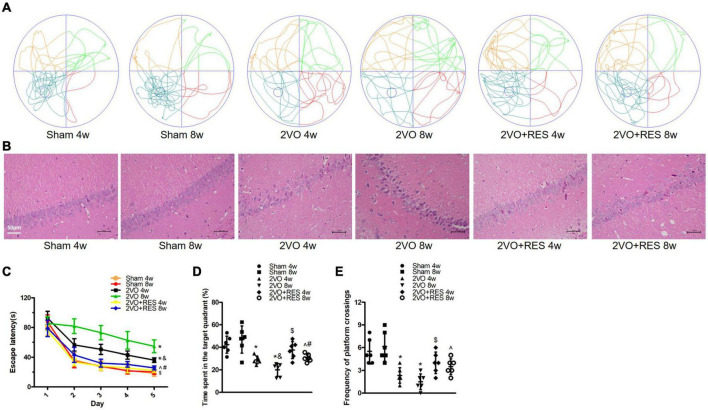
Resveratrol (RES) alleviates cognitive deficits and pathologic changes in the hippocampal CA1 region at 4 and 8 weeks after bilateral common carotid artery occlusion (2VO). **(A)** Swimming path of rats on day 6 in different groups. **(B)** Representative images of Hematoxylin–eosin staining in the hippocampal CA1 region (× 200, scale bar, 50 μm). **(C)** Escape latency from day 1 to day 5. **(D)** Probe trial on day 6. **(E)** Frequency of platform crossings. Morris water maze test, *n* = 6 per group; Hematoxylin–eosin staining, *n* = 3 per group. *p < 0.05 vs. Sham; ^&^*p* < 0.05, 2VO 8w group vs. 2VO 4w group; ^#^*p* < 0.05, 2VO + RES 8w group vs. 2VO + RES 4w group; ^$^*p* < 0.05, 2VO + RES 4w group vs. 2VO 4w group; ^*p* < 0.05, 2VO + RES 8w group vs. 2VO 8w group. Data are presented as mean ± SD.

The effect of RES on neurological impairments was evaluated through the Morris water maze test of rats at 4 and 8 weeks after 2VO. The escape latency of the control rat significantly decreased from days 1 to 5, reflecting normal learning ability. On day 6, the control rat spent significantly longer time in the escape platform quadrant, indicating normal retrieval. As displayed in Figure 2, a statistical analysis showed that CCH induced a significant increase in the escape latency of the rat compared with that found for the control animals (2VO 4w vs. Sham 4w group, 2VO 8w vs. Sham 8w group, *P* < 0.05; Figure 2C), and this increase indicates an impairment in the spatial learning ability of the rat. Moreover, the escape latency of the 2VO 8w rat was longer than that of the 2VO rat sacrificed at 4 weeks (2VO 8w vs. 2VO 4w group, *P* < 0.05; Figure 2C), indicating a progressive impairment in learning over time after CCH. Treatment with RES at 20 mg/kg/day significantly reversed the CCH-induced increase in escape latency (2VO + RES 8w vs. 2VO 8w, 2VO + RES 4w vs. 2VO 4w group, *P* < 0.05; Figure 2C). In the probe test, the CCH animals spent a significantly shorter time in the target quadrant and had fewer platform crossings than the control rat, which also indicates an impairment in retrieval (*P* < 0.05; Figures 2D,E). Rat in the 2VO 8w group spent a decreased amount of time in the quadrant containing the escape platform than those belonging to the 2VO 4w group. RES treatment increased the time spent in the target quadrant and had a higher frequency of platform crossings at both time points (2VO + RES 8w vs. 2VO 8w group, 2VO + RES 4w vs. 2VO 4w group, all *P* < 0.05; Figures 2D,E). In summary, RES restored spatial memory and learning impairments of rats with CCH.

### Resveratrol decreases the pathological changes in the hippocampal CA1 region induced by chronic cerebral hypoperfusion

The protective effect of RES was also assessed based on neuronal death in the hippocampus. The results showed that RES treatment partly reduced pyknosis of the cytoplasm and neuronal shrinkage and loss, as determined by HE staining (Figure 2B).

## Resveratrol reduces white matter damage in corpus callosum after chronic cerebral hypoperfusion

Because white matter damage contributes to cognitive impairment following CCH, we examined the damage to the myelin sheath in the corpus callosum by measuring the loss of myelin *via* Luxol fast blue staining and by assessing the loss of myelin basic protein (MBP).

**FIGURE 3:**
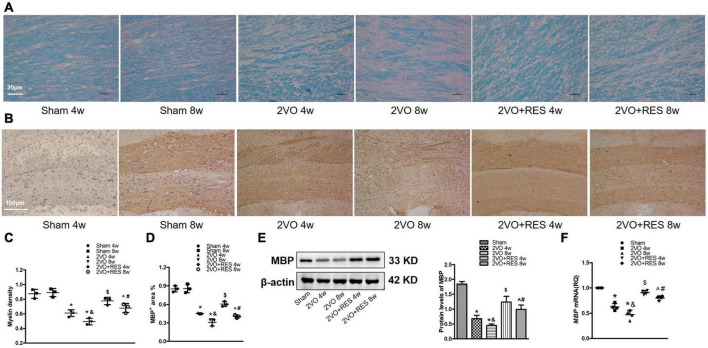
Resveratrol (RES) attenuates white matter damage in corpus callosum after bilateral common carotid artery occlusion (2VO). **(A)** Luxol fast blue (LFB) staining in the corpus callosum at 4 and 8 weeks after 2VO (*n* = 3 per group, ×400, scale bar, 20 μm). **(B)** Representative immunostaining for myelin basic protein (MBP) staining in the corpus callosum in different groups (*n* = 3 per group, ×100, scale bar, 100 μm). **(C)** Myelin density in each group is presented by the percentage of LFB-stained area in a total area of the corpus callosum. **(D)** Quantitation of the percentage of MBP positive area in the corpus callosum. **(E)** Left, western blots of MBP from white matter extract. right, quantification of western blot analysis of MBP after 2VO. β-actin was used as a loading control (*n* = 3 per group). **(F)** Quantification of mRNA levels of MBP. GAPDH was used as an internal control (*n* = 4 per group). *p < 0.05 vs. Sham; ^&^*p* < 0.05, 2VO 8w group vs. 2VO 4w group; ^#^*p* < 0.05, 2VO + RES 8w group vs. 2VO + RES 4w group; ^$^*p* < 0.05, 2VO + RES 4w group vs. 2VO 4w group; ^*p* < 0.05, 2VO + RES 8w group vs. 2VO 8w group. Data are presented as mean ± SD.

Compare with corresponding Sham groups, the rat in 2VO groups showed reduced myelin density (Figures 3A,C) and percentage of MBP positive area in the corpus callosum (Figures 3B,D), while RES treatment partially restored myelin density and percentage of area positive for MBP at 4 and 8 weeks. Our preliminary experiments have shown that, compared with the Sham group treated with vehicle for 4 weeks, there was no significant difference in escape latency, amount of time spent in the target quadrant, frequency of platform crossings, neuronal death, and myelin density when the vehicle was administered for 8 weeks in Sham rat after CCH (*P* > 0.05). Accordingly, we displayed one Sham group in subsequent qRT-PCR and Western-blotting studies.

The 2VO model rat exhibited decreased MBP protein and mRNA levels relative to the Sham group (2VO 4w and 2VO 8w vs. Sham, all *P* < 0.05; Figures 3E,F). Additionally, demyelination was more severe in the 2VO 8w group compared to the 2VO 4w group (2VO 8w vs. 2VO 4w group, *P* < 0.05; Figures 3C–F), reflecting the progressive disruption of myelin over time following CCH. As expected, RES significantly elevated the expression of MBP at 4 and 8 weeks (2VO + RES 8w vs. 2VO 8w, 2VO + RES 4w vs. 2VO 4w group, all *P* < 0.05; Figures 3D–F). The results presented thus far suggest that RES protects against white matter damage.

### Resveratrol reduces the mRNA levels of inflammatory mediators

To assess whether the protective effect of RES against CCH injury is related to its anti-inflammatory properties, we next examined classical neuroinflammatory responses following CCH. The gene expression of pro-inflammatory cytokines, tumor necrosis factor-alpha (TNF-α), the chemokine CXCL10, and adhesion molecules intercellular adhesion molecule 1 (ICAM-1) and vascular adhesion molecule 1 (VCAM-1), and anti-inflammatory cytokine IL-10 were assessed in the white matter of rat at 4- and 8-weeks post-surgery. The gene expression of ICAM-1 and VCAM-1 was also assessed in the hippocampus of rats.

**FIGURE 4:**
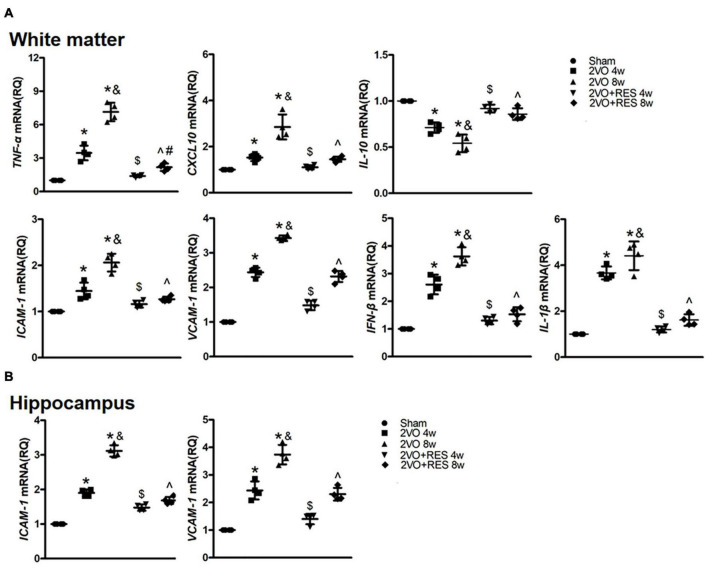
The expression of pro- and anti-inflammatory genes. **(A)** Quantification of mRNA levels of TNF-α, CXCL10, IL-10, ICAM-1, VCAM-1, IFN-β, and IL-1β in white matter tissues. GAPDH was used as an internal control (*n* = 4 per group). **(B)** Quantification of mRNA levels of ICAM-1 and VCAM-1 in hippocampus tissues. GAPDH was used as an internal control (*n* = 4 per group). *p < 0.05 vs. Sham; ^&^*p* < 0.05, 2VO 8w group vs. 2VO 4w group; #*p* < 0.05, 2VO + RES 8w group vs. 2VO + RES 4w group; ^$^*p* < 0.05, 2VO + RES 4w group vs. 2VO 4w group; ^*p* < 0.05, 2VO + RES 8w group vs. 2VO 8w group. Data are presented as mean ± SD.

Chronic cerebral hypoperfusion induced a robust increase in the mRNA expression of TNF-α, CXCL10, ICAM-1, and VCAM-1 in white matter (2VO 4w and 2VO 8w vs. Sham group, *P* < 0.05, respectively; Figure 4A). In addition, the mRNA levels of TNF-α, and CXCL10 in the 2VO 8w group were higher than those found in the 2VO 4w group (2VO 8w vs. 2VO 4w group, *P* < 0.05, Figure 4A). The analysis demonstrated a significant reduction of TNF-α, CXCL10, ICAM-1, and VCAM-1 mRNA expression in RES-treated rats (2VO + RES 8w vs. 2VO 8w, 2VO + RES 4w vs. 2VO 4w group, *P* < 0.05, Figure 4A). Similar patterns were observed in mRNA expression of ICAM-1 and VCAM-1 in the hippocampus (Figure 4B). RES treatment after 4- and 8 weeks resulted in increased expression of IL-10 compared with the levels found in the 2VO group (2VO + RES 8w vs. 2VO 8w, 2VO + RES 4w vs. 2VO 4w group, *P* < 0.05; Figure 4A).

### stimulator of interferon genes, TANK-binding kinase 1, and interferon regulatory factor 3 expressions are involved in chronic cerebral hypoperfusion and inhibited by H-151 or resveratrol in bilateral common carotid artery occlusion cerebral tissue

To examine whether STING, TBK1, and IRF3 are needed for the neuroinflammatory responses induced by CCH and whether the inhibitory effect of RES on cerebral inflammatory responses is related to the expression of STING, TBK1, and IRF3, we examined the temporal patterns of STING, TBK1 and IRF3 expression in the hippocampus and white matter of 2VO rat.

**FIGURE 5:**
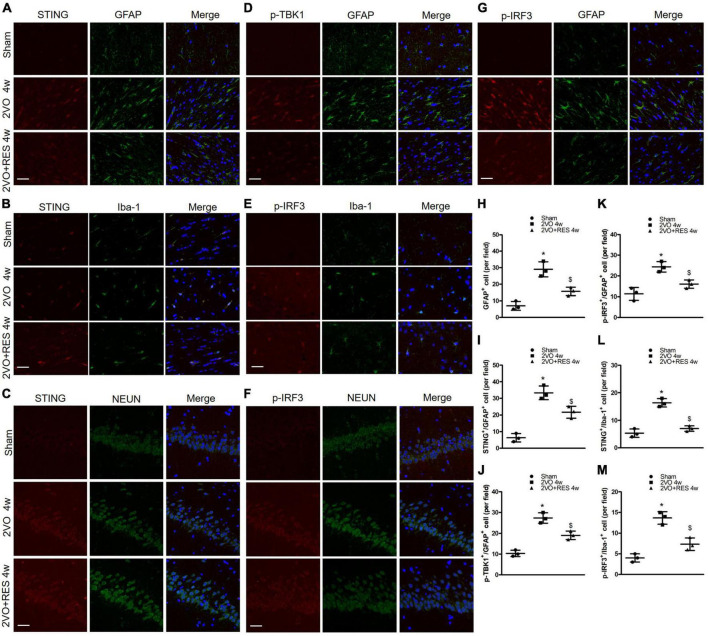
**(A,D,G)** Representative images of astrocytes (GFAP-positive cells, green) and stimulator of interferon genes (STING) (red), p-TANK-binding kinase 1 (p-TBK1; red), p-interferon regulatory factor 3 (p-IRF3; red) immunofluorescence double-labeling in white matter at 4 weeks after bilateral common carotid artery occlusion (2VO). **(C,F)** shows immunofluorescence of STING (red) and p-IRF3(red) expression were colocalized with neurons (NEUN-positive cells, green) in the hippocampus. **(B,E)** Representative immunofluorescent images of microglia (Iba-1-positive cells, green) and STING (red), p-IRF3(red) immunofluorescence double-labeling in white matter. (×400, Blue, DAPI, Scale bar, 20 μm, *n* = 3 per group) **(H)** Quantitative analysis of the number of GFAP^+^ cells. **(I–K)** Quantitation of the number of STING^+^/GFAP^+^ cells, p-TBK1^+^/GFAP^+^ cells, and p-IRF3^+^/GFAP^+^ cells in different groups. **(L,M)** Quantitation of the number of STING^+^/Iba-1^+^ cells, p-IRF3^+^/Iba-1^+^ cells. *p < 0.05 vs. Sham; ^$^*p* < 0.05, 2VO + RES 4w group vs. 2VO 4w group. Data are presented as mean ± SD.

Double immunofluorescent staining showed that STING- and phospho-IRF3-positive cells were abundantly colocalized with NEUN-positive cells in the hippocampus (Figures 5C,F). STING and phospho-IRF3 were colocalized with Iba-1 positive cells in white matter (Figures 5B,E). STING, phospho-TBK1, and phospho-IRF3 were also colocalized with GFAP-positive cells in white matter (Figures 5A,D,G). This suggests that neurons, microglia, and astrocytes are involved in the STING-mediated response after CCH.

Furthermore, low numbers of GFAP-positive cells were detected in the white matter of the Sham group. In contrast, the 2VO group exhibited a significantly ascending number of positive GFAP marked astrocytes (2VO 4w group vs. Sham, *P* < 0.05, Figure 5H). RES treatment suppressed 2VO-induced reactive astrogliosis in white matter at 4 weeks after 2VO. Compared with the Sham group, the number of STING positive and Iba-1 positive microglia ascends significantly in the white matter of the 2VO group, and the number of STING positive microglia was decreased in the white matter of the RES-treated group at 4 weeks after 2VO (2VO 4w group vs. Sham, 2VO + RES 4w group vs. 2VO 4w group, *P* < 0.05, Figures 5B,L). The same trend was found in a number of p-IRF3 positive microglia (2VO 4w group vs. Sham, 2VO + RES 4w group vs. 2VO 4w group, *P* < 0.05, Figures 5E,M). Compared to the sham group, the 2VO group showed an increased number of STING and GFAP, p-TBK1 and GFAP, p-IRF3 and GFAP double-positive cells (2VO 4w group vs. Sham, *P* < 0.05, Figures 5I–K). The number of these cells was reduced in the 2VO group treated with RES. (2VO + RES 4w vs. 2VO 4w group, *P* < 0.05, Figures 5I–K).

**FIGURE 6:**
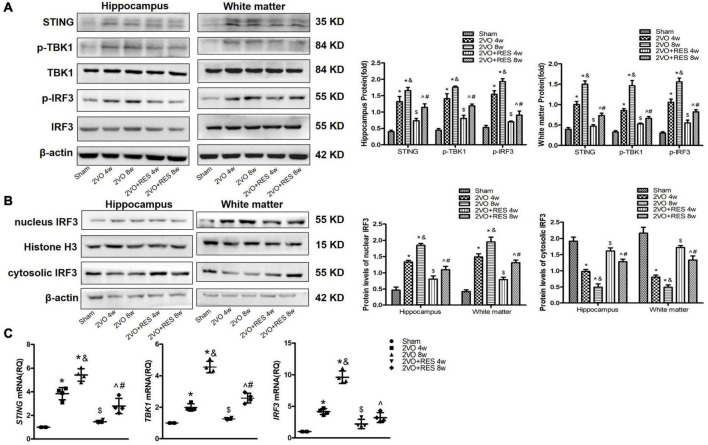
Increased expression of stimulators of type 1 interferon gene (STING), p-TANK-binding kinase 1 (p-TBK1), and p-interferon regulatory factor 3 (p-IRF3) in bilateral common carotid artery occlusion (2VO) Rats. The expression of STING, p-TBK1, and p-IRF3 was decreased in 2VO rats treated with resveratrol (RES). 2VO promotes the nuclear translocation of IRF3. RES treatment counteracted the nuclear translocation of IRF3. **(A)** Western blot and quantitative analysis of STING, TBK1, IRF3, p-TBK1, and p-IRF3 at weeks 4 and 8 in hippocampus and white matter extract after 2VO. β-actin was used as an internal control (*n* = 3 per group). **(B)** Western blot analysis of nucleus and cytosolic IRF3 in white matter and hippocampus in different groups after 2VO. β-actin was used as an internal control (*n* = 3 per group). **(C)** mRNA levels of STING, TBK1, and IRF3 in white matter were detected by qRT-PCR. GAPDH was used as an internal control (*n* = 4 per group). *p < 0.05 vs. Sham; ^&^*p* < 0.05, 2VO 8w group vs. 2VO 4w group; ^#^*p* < 0.05, 2VO + RES 8w group vs. 2VO + RES 4w group; ^$^*p* < 0.05, 2VO + RES 4w group vs. 2VO 4w group; ^*p* < 0.05, 2VO + RES 8w group vs. 2VO 8w group. Data are presented as mean ± SD.

Our data show that CCH increased hippocampal expression of STING protein in 2VO rat compared to Sham rat (2VO 4w and 2VO 8w vs. Sham group, *P* < 0.05, respectively; Figure 6A); however, STING protein expression was dramatically decreased after treatment with RES following 2VO (2VO + RES 8w vs. 2VO 8w, 2VO + RES 4w vs. 2VO 4w group, all *P* < 0.05; Figure 6A). Similar to STING, phospho-TBK1 and phospho-IRF3 protein expressions in the hippocampus were also increased after 2VO compared to the Sham group (2VO 4W and 2VO 8W vs. Sham group, all *P* < 0.05, respectively; Figure 6A). CCH-mediated increase of phospho-TBK1 and phospho-IRF3 expression in the hippocampus was considerably decreased by RES treatment following CCH. Moreover, the vehicle-treated CCH rat sacrificed at 8 weeks exhibited increased STING, p-TBK1, and p-IRF3 protein expression levels than rats of the 2VO 4w group (2VO 8w vs. 2VO 4w group, *P* < 0.05, Figure 6A). Similar protein expression patterns were observed in white matter at 4- and 8 weeks post-injury (Figure 6A).

Similarly, qRT-PCR assays identified increased mRNA expression of STING, TBK1, and IRF3 in 2VO rat treated with vehicle as compared to the Sham rat, and the expression of these genes was decreased in 2VO rat treated with RES at 4 and 8 weeks after 2VO (*P* < 0.05, Figure 6C).

To further clarify the specificity of the STING pathway in the rat model of CCH, we administered rats with the STING inhibitor H-151. The protein expression of the STING axis was increased in white matter and hippocampus of rats subjected to CCH for 4 weeks, while H-151 potently inhibited STING expression, as evidenced by reduction of TBK1 and IRF3 phosphorylation without affecting respective controls (Supplementary Figure 1). In addition, ELISA analyses found that the concentration of 2′3′-cGAMP was markedly elevated at 4 weeks after 2VO than that in the Sham group, demonstrating that cGAS was activated following CCH (Supplementary Figure 2). Together, these results indicate that the cGAS/STING pathway was induced by CCH.

### Location and translocation of p-interferon regulatory factor 3 in neurons, astrocytes, and microglia after chronic cerebral hypoperfusion

We examined the expression of p-IRF3 in neurons, astrocytes, and microglia 4 weeks after 2VO through immunofluorescence analyses. As shown in Figures 5E–G, in the Sham group, p-IRF3 was detectable in a few neurons and almost no astrocytes or microglia, and most cells labeled by p-IRF3 showed staining only in the cytoplasm. In the 2VO group, the expression of p-IRF3 was found in neurons, microglia, and astrocytes, and p-IRF3 was localized in both the cytoplasm and the nucleus. Compared with the results obtained for the model group, the number of cells positive for p-IRF3 in the RES group was reduced at 4 weeks after 2VO (2VO + RES 4w vs. 2VO 4w group, *P* < 0.05, Figures 5K,M). Moreover, some of the cells labeled with p-IRF3 showed staining only in the cytoplasm (Figures 5E–G).

As indicated in Figure 6B, we also detected the protein expression of IRF3 in the nucleus and found that CCH could significantly induce the nuclear translocation of IRF3 without affecting the total protein expression of IRF3, whereas RES treatment counteracted the nuclear translocation of p-IRF3 (2VO + RES 8w vs. 2VO 8w, 2VO + RES 4w vs. 2VO 4w group, *P* < 0.05, Figure 6B).

### Stimulator of interferon genes/TANK-binding kinase 1/interferon regulatory factor 3 pathway mediated type-I interferon is suppressed in cerebral tissue of resveratrol-treated bilateral common carotid artery occlusion groups

We then sought to determine whether the STING/TBK1/IRF3 signaling mediates the expression of proinflammatory cytokines in response to CCH and whether RES inhibits these proinflammatory cytokines. Previous studies have identified IFN-β as one of the main genes responding to STING-dependent IRF3 activation (Mathur et al., 2017). As shown in Figure 4A, the mRNA expression of IFN-β and IL-1β in white matter were markedly induced by CCH at 4 and 8 weeks (2VO 4W and 2VO 8W vs. Sham group, *P* < 0.05, respectively; Figure 4A). However, these results were markedly restrained in the RES-treated CCH group than in the model group (2VO + RES 8w vs. 2VO 8w, 2VO + RES 4w vs. 2VO 4w group, all *P* < 0.05; Figure 4A). Moreover, results revealed significant parallel alterations in the mRNA expression levels of IRF3 and IFN-β genes.

These results indicate that the upregulation of these proinflammatory cytokines may be induced by STING/TBK1/IRF3 signaling in CCH tissue. Decreases of the IFN-β and IL-1β mRNA in RES-treated rats were likely due to inhibition of the STING/TBK1/IRF3 signaling.

### Activation of stimulator of interferon genes/TANK-binding kinase 1/interferon regulatory factor 3 signaling may regulate the microglial morphology

Microglia are the primary immune cells enriched in brain tissue. These cells respond to insults by changing their morphology and cytokine production. Specifically, when activated, microglial cells change their morphology to exhibit larger nuclei and shorter processes.

**FIGURE 7:**
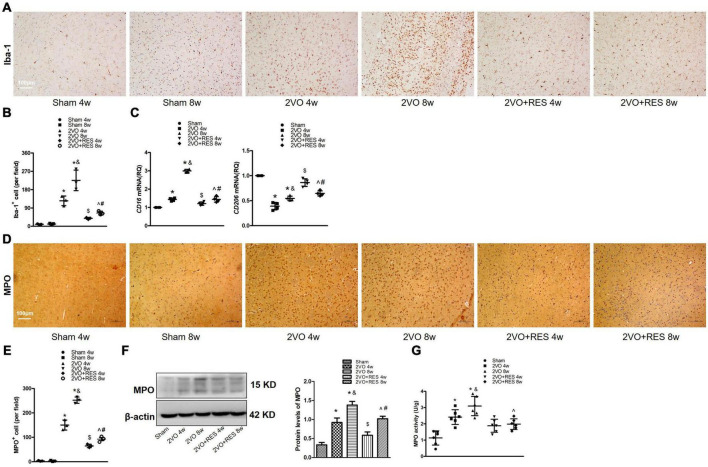
Increased microglial activity and neutrophil expression at 4 and 8 weeks after 2VO. Resveratrol (RES) treatment inhibits gliosis and neutrophil expression in white matter induced by bilateral common carotid artery occlusion (2VO). **(A)** Assessment of microglial proliferation and activation. Representative images of Iba-1-stained white matter in each group (*n* = 3 per group, ×100, scale bar, 100 μm). **(B)** Quantitation of Iba-1 positive cells in white matter. **(C)** Quantitative analysis of mRNA levels of CD16 and CD206. GAPDH was used as an internal control (*n* = 4 per group). **(D)** Representative images of Immunohistochemical staining for myeloperoxidase (MPO) in the white matter of rats after 2VO (*n* = 3 per group, ×100, scale bar, 100 μm) **(E)** Quantitative analysis of the number of MPO-positive cells. **(F)** Western blot and quantification of MPO protein expression in white matter. β-actin was used as an internal control (*n* = 3 per group). **(G)** MPO activity was examined by ELISA in white matter in different groups at 4 and 8 weeks after 2VO (*n* = 6 per group). *p < 0.05 vs. Sham; ^&^*p* < 0.05, 2VO 8w group vs. 2VO 4w group; ^#^*p* < 0.05, 2VO + RES 8w group vs. 2VO + RES 4w group; ^$^*p* < 0.05, 2VO + RES 4w group vs. 2VO 4w group; ^*p* < 0.05, 2VO + RES 8w group vs. 2VO 8w group. Data are presented as mean ± SD.

To further investigate the effect of RES on microglia following CCH, we performed Immunohistochemical staining for the microglial marker Iba-1 in white matter after CCH and showed the morphology of Iba1 positive glia in the white matter of rats at 4 and 8 weeks after sham or 2VO operation (Figure 7A). As expected, a number of cells labeled with Iba-1 were elevated in the 2VO group (2VO 4W and 2VO 8W vs. Sham group, *P* < 0.05, respectively; Figure 7B) but partially suppressed by RES treatment at both time points (2VO + RES 8w vs. 2VO 8w, 2VO + RES 4w vs. 2VO 4w group, all *P* < 0.05; Figure 7B). In addition, the model rat sacrificed 4 weeks after 2VO showed less severe gliosis than those sacrificed at 8 weeks (2VO 8w vs. 2VO 4w group, *P* < 0.05, Figure 7B). A qRT-PCR analysis of the white matter extract showed that the M1 marker CD16 was increased after CCH but significantly decreased by RES treatment, whereas the M2-type genes (CD206 and IL-10 genes) were increased by RES at both post-surgery time points (2VO + RES 8w vs. 2VO 8w, 2VO + RES 4w vs. 2VO 4w group, *P* < 0.05, Figures 4A, 7C).

These results suggest that glia in the white matter was activated after 2VO. RES treatment shifts the microglial polarization toward M2, reduced microglial proliferation in the white matter after CCH, and STING/TBK1/IRF3 axis may involve in this process.

### Resveratrol prohibits neutrophil infiltration in chronic cerebral hypoperfusion

Because we found the elevated expression of ICAM-1, VCAM-1, TNF-α, and IL-1β, we further detected the alterations in myeloperoxidase.

Specifically, we quantified the number of MPO-positive cells, MPO activity, and MPO expression in white matter. Immunostaining of MPO confirmed the neutrophil presence in white matter after 2VO (Figure 7D). By week 8, neutrophils infiltrated white matter (Figure 7D). As indicated, the number of MPO-positive cells was decreased in the RES groups at 4 and 8 weeks after 2VO (2VO + RES 8w vs. 2VO 8w, 2VO + RES 4w vs. 2VO 4w group, *P* < 0.05, Figure 7E). A Western blot analysis demonstrated that hypoperfusion caused an increase in MPO expression in white matter at 4 and 8 weeks after 2VO and that RES treatment attenuated these increases (2VO 4W and 2VO 8W vs. Sham group, 2VO + RES 8w vs. 2VO 8w, 2VO + RES 4w vs. 2VO 4w group, all *P* < 0.05, Figure 7F).

Myeloperoxidase activity is a good indicator of inflammation and neutrophil accumulation and can be quantified *via* the MPO activity assay. As shown in Figure 7G, MPO activity in white matter was increased at 4 and 8 weeks after 2VO and was also significantly reduced in the rat belonging to the RES group at 8 weeks after 2VO. Here, we observed that, other than causing microglia transformation toward an anti-inflammatory phenotype, RES is capable to dampen neuroinflammatory response by reducing the migration of periphery neutrophils into the CNS.

### Resveratrol treatment decreases chronic cerebral hypoperfusion-induced endoplasmic reticulum stress-related markers

**FIGURE 8:**
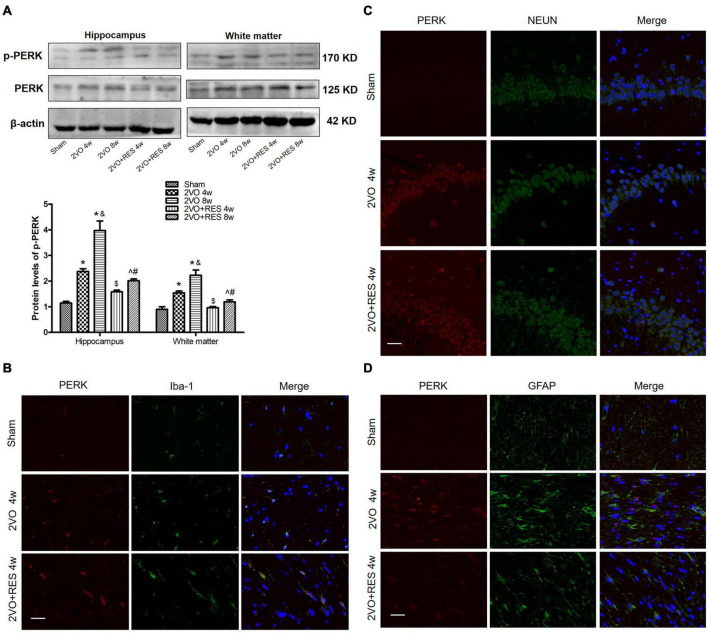
Aberrant endoplasmic reticulum (ER)-stress in the bilateral common carotid artery occlusion (2VO) model is partially prohibited by resveratrol (RES). **(A)** The protein levels of PKR-like ER kinase (PERK) and p-PERK in white matter and hippocampus of different groups were measured by Western blotting. β-actin was used as an internal control (*n* = 3 per group). The presence of p-PERK (red) in microglia **(B)**, neurons **(C)**, and astrocytes **(D)** was tested by immunofluorescence (× 400, Blue, DAPI, *n* = 3 per group). *p < 0.05 vs. Sham; ^&^*p* < 0.05, 2VO 8w group vs. 2VO 4w group; ^#^*p* < 0.05, 2VO + RES 8w group vs. 2VO + RES 4w group; ^$^*p* < 0.05, 2VO + RES 4w group vs. 2VO 4w group; ^*p* < 0.05, 2VO + RES 8w group vs. 2VO 8w group. Data are presented as mean ± SD.

Our immunofluorescence results showed that PERK was located in microglia (Figure 8B), neurons (Figure 8C), and astrocytes (Figure 8D). In addition, the protein expression levels of p-PERK were significantly elevated in 2VO groups but deregulated in the RES-treated groups (2VO 4w and 2VO 8w vs. Sham group, 2VO + RES 8w vs. 2VO 8w, 2VO + RES 4w vs. 2VO 4w group, all *P* < 0.05, Figure 8A). Therefore, our CCH model rat experienced ER stress, and RES treatment inhibited this ER stress. As PERK and STING are both located in neurons, microglia and astrocytes, and PERK protein expression was observed correlations with STING protein expression, STING was probably activated by ER stress in this 2VO model.

## Discussion

This study provides the first demonstration that (1) Activation of the STING/TBK1/IRF3 signaling may be triggered by ER stress during CCH in rats; (2) Increased neutrophilic recruitment, gliosis, and generation of key proinflammatory mediators are positively correlated with activation of STING/TBK1/IRF3 signaling; (3) Enhanced STING expression, phosphorylation of TBK1 and IRF3 contributes to the progressive cognitive decline and white matter damage observed in CCH over time; (4) Activation of STING/TBK1/IRF3 signaling and subsequent neuroinflammatory processes are markedly attenuated in RES-treated rat after CCH; and (5) RES reduces the number of activated microglia, astrocyte and alleviates myelin loss after CCH.

Neuroinflammation is known to be a key instigator of detrimental injury after VaD. The identification of key molecules that regulate neuroinflammatory processes could pinpoint a potential therapeutic target that could result in improved patient outcomes following VaD (O’Brien and Thomas, 2015; Lecordier et al., 2021). STING was initially characterized as a sensor of cytosolic DNA that promotes survival after infection (Moretti et al., 2017). Recent findings suggest that reduced STING/IRF3 signaling is associated with an attenuated neuroinflammatory response and subsequent neuroprotection in neurodegeneration (Quek et al., 2017; Hou et al., 2021), acute cerebral ischemia/reperfusion (I/R) injury (Li Q. et al., 2020; Liao et al., 2020) and TBI (Barrett et al., 2020). We provide the first demonstration that the STING/TBK1/IRF3 axis is robustly activated in a CCH rat model. Expression of STING, TBK1, and IRF3 phosphorylation, as well as the nuclear translocation of IRF3, were significantly elevated in the hippocampus and white matter of rats with CCH, and this elevation was paraleled by upregulated levels of key proinflammatory cytokines and increased neutrophil infiltration. Here, we found that STING, p-TBK1, and p-IRF3 were widely expressed in neurons, microglia, and astrocytes in brain tissues after CCH, corroborating previous findings that the STING pathway is activated in microglia and leads to IR-induced neuroinflammation and brain injury (Liao et al., 2020). STING also drives astrocyte and microglial reactivity in the TBI model (Barrett et al., 2020). STING/TBK1/IRF3 axis is found to be activated in neurons, which induces inflammatory cytokine production and leads to neuronal death in a rat model of ataxia-telangiectasia (Quek et al., 2017). Furthermore, we provide the first confirmation that STING/TBK1/IRF3 axis is possibly associated with deteriorating neuronal death, gliosis, white matter damage, and impairments in learning and memory after CCH in a time-dependent manner. Therefore, we infer from these findings that STING/TBK1/IRF3 axis contributes to the detrimental neuroinflammatory environment in a CCH animal model of VaD.

However, a study has suggested that stimulation of the STING/IRF3 pathway induces a reduction in neuroinflammation in a transgenic mouse model of Alzheimer’s disease (Xu et al., 2019). Another study showed that STING-KO mice display exacerbated endogenous retrovirus activation along with pronounced hippocampal neuron loss and gliosis (Sankowski et al., 2019). These discrepancies may be due to the use of different animal species and disease models and to variations in the sampled parts and time points. The biological effect of STING signaling exhibits considerable heterogeneity, varies according to the acute/chronic course, and shows low/high intensity. Thus, more detailed studies of the molecular mechanism underlying STING signaling and its regulators are needed.

The mammalian IRFs family includes transcription factors that induce type I IFNs (IRF3, IRF7), propagate type I IFN responses (IRF1, IRF4, and IRF5), and play a central role in innate immunity (Günthner and Anders, 2013). IRF-1 resists cognitive decline under normal conditions, but no obvious effect on cognition was observed in a bilateral common carotid artery stenosis mouse model (Mogi et al., 2018). The transcription factor IRF3 is constitutively expressed in all cell types and is essential for the induction of the IFN genes, IFN-α and IFN-β (Akira et al., 2006). A previous study found that mice lacking IRF3 exhibit protection after acute cerebral I/R injury (Marsh et al., 2009). However, a subsequent study showed that both the mRNA and protein levels of IRF3 are significantly increased in rats after transient global cerebral ischemia injury (Cui et al., 2013). Similar to the above-mentioned study, IRF3 ablation in rats decreased cerebral I/R-induced inflammation and the expression of some proinflammatory genes, such as IL-1β, TNF-α, and ICAM-1 (Li L. et al., 2016). Therefore, IRF3 may exhibit functions during ischemic stroke in rats that is different from those found in mice. Consistent with these studies, this study revealed that IRF3 was activated in a 2VO rat model, accompanied by elevated expression of proinflammatory genes, such as IFN-β and IL-1β. Based on our immunofluorescence results, phospho-IRF3 was widely expressed in neurons, microglia, and astrocytes in brain tissues, and after injury, IRF3 was transported to the nucleus, in accordance with previous studies (Li et al., 2019).

Our previous study validates that ER stress is one of the major contributors to secondary injuries during hypoperfusion (Niu et al., 2019). The present results revealed that PKR-like ER kinase (PERK) is induced in neurons, microglia and astrocytes, in agreement with former studies (Guthrie et al., 2016; Liao et al., 2016). We identified that an increase in PERK phosphorylation in the hippocampus and white matter initiates ER-stress in rats after 2VO surgery. We demonstrated that ER stress induces tissue damage in hippocampus and white matter regions, STING is abundantly present in the same areas, and PERK protein expression was correlated with STING protein expression, which implies that ER stress might modulate the initiation of STING after CCH. Nevertheless, we are aware that the STING signaling pathway may be triggered by damage-associated molecular patterns other than ER stress after CCH. The generation of 2′3′-cGAMP demonstrates the engagement of cGAS in this CCH model. Thus, we cannot exclude other potential activators of STING, in particular cytosolic double-stranded DNA, which was recognized by cGAS. Microglial and astrocyte reactivity is a generally acknowledged neuroinflammatory feature after CCH (Hase et al., 2018). Aberrant M1-type microglial activation participates in white matter injury after cerebral hypoperfusion (Zhang L. Y. et al., 2020), and M2-type microglia exhibit increased phagocytosis of myelin debris and secretion of trophic factors that stimulate oligodendrocytes to facilitate remyelination in white matter (Dudvarski Stankovic et al., 2016; Lee et al., 2019). In this study, we found that increased expression of Iba-1 positive cell staining and an altered microglial phenotype after CCH was correlated with activation of the STING cascade. We also detected elevated GFAP expression 4 weeks after CCH (Figure 5H). White matter damage was consistent with the expression of microglia and astrocytes. RES-treated rats displayed diminished levels of microglial activation and astrocyte hypertrophy. In addition, RES-treated rats showed upregulation of CD206 mRNA and downregulation of CD16 mRNA, which suggested that RES intervention potentially promotes microglial polarization toward the M2 phenotype. Reductions in glial reactivity and white matter damage after CCH may contribute to the neuroprotective effects observed in the RES group, and these effects may be related to the inhibition of STING/TBK1/IRF3 signaling. Collectively, the results indicate that STING/TBK1/IRF3 signaling contributes to neuroinflammation partly by driving astrocyte and microglial reactivity. RES suppressed inflammation following 2VO by regulating microglial polarization and reduction in the release of inflammatory mediators, partly through inhibition of the STING/TBK1/IRF3 axis. These effects were consistent with those detailed in a recent review, which described that RES might mediate the regulation of microglial phenotypes and functions to control neuroinflammation in neural degenerative diseases and TBI (Maurya et al., 2021). A report also illustrated that RES enhances anti-inflammatory and decreases inflammatory cytokines by affecting the signaling pathways in microglia and astrocytes, and increases oligodendrocyte survival in the stroke model (Ghazavi et al., 2020). However, a recent study showed that STING activation reduces microglial reactivity and confers protection in multiple sclerosis animal models (Mathur et al., 2017). This dual function of STING in regulating microglial reactivity may be attributed to the different disease models used in the studies.

Chronic inflammation is one of the major factors in the pathogenesis of VaD (Sinha et al., 2020). In CCH, reactive microglia and astrocytes release proinflammatory cytokines, chemokines, and inflammatory mediators, which results in neurotoxicity (Rosenberg et al., 2014). Neuroinflammation contributes to white matter lesions and neuronal loss and thereby results in compromised learning and memory dysfunction (Farkas et al., 2007). Inflammatory chemokines or cytokines stimulate the expression of adhesion molecules, which leads to the extravasation of neutrophils into the brain parenchyma (Konsman et al., 2007; Kreuger and Phillipson, 2016). During CCH, TNF-α, IL-1β, IL-6, VCAM-1, and ICAM-1 are well-established molecules that mediate the initiation of the inflammatory response and appear to exacerbate the hypo-perfused injury. In our study, a certain number of factors related to the inflammatory response, such as inflammatory cytokines (IL-1β and TNF-α), chemokines (CXCL10), and inflammatory mediators (ICAM-1, VCAM-1), were found to be partially reduced by RES. This finding is in line with an earlier study, which demonstrated that RES inhibits the expression of inflammation-associated genes, such as IFN-β, by reducing the kinase activity of TBK1, inhibiting IRF3 and NF-κB activation, and blocking the binding of active IRF3 to target gene promoters in RAW264.7 cells (Youn et al., 2005).

Chronic cerebral hypoperfusion induces peripheral neutrophil recruitment, which leads to tissue damage and neurological deficits (Wu et al., 2020). The brain level of MPO was measured as a marker of neutrophil infiltration in the CCH rat model (Shin et al., 2008). We found that CCH increased neutrophil infiltration into the brain, as evidenced by increased MPO activity and expression. The level of MPO was in agreement with the results found for the STING/TBK1/IRF3 axis and pro-inflammatory molecules. This finding was in keeping with previous research that IRF3-knockout mice show abrogated neutrophil recruitment and reduced MPO activity in the pancreas (Akbarshahi et al., 2011). Altogether these results support a pathologic role of STING/TBK1/IRF3 cascade on inflammatory conditions. Our results indicate that RES modifies the chronic inflammatory processes of neutrophil recruitment following cerebral hypoperfusion, which agrees with previous reports that RES reduces neutrophil activation in a dose-dependent manner to attenuate acute lung injury (Tsai et al., 2019). Moreover, RES reduces MPO expression and attenuates brain damage in permanent focal cerebral ischemia (Lei and Chen, 2018). Our results indicate that the numbers of neutrophils and microglia were reduced and the activation of microglia was attenuated by RES treatment (Figure 7). RES can efficiently inhibit the migration of both resident microglia and peripheral neutrophils toward damaged tissue and the proliferation of microglia.

There have been several studies that reported that RES has an ameliorative effect on impairments in spatial learning and memory and CNS pathology associated with CCH. Anastácio et al. (2014) found that RES (20 mg/kg, intraperitoneal) treatment to Wistar Rats after modified 2VO protocol significantly attenuated pyramidal cell death in the hippocampus CA1 region, along with maintaining the expression of nerve growth factor (NGF) until 45 days after surgery. Another study has proposed that four weeks of pretreatment with RES (40 mg/kg, intraperitoneal) alleviates LTP inhibition and dendritic spine loss, as well as the decreased expression of synaptic proteins, PSD95, NMDA receptor 2A/B (NR2A/B), and PSD93 induced by CCH. Four weeks after 2VO surgery, RES also increased the activity of protein kinase A (PKA), cAMP levels, and the phosphorylation of cAMP-responsive element-binding (CREB) protein, a critical transcriptional factor in the memory process (Li H. et al., 2016). A recent study indicates, that after RES (50 mg/kg, intragastrical) treatment for 21 consecutive days, reduced pathological damage and brain damage markers S-100β and NSE in the frontal cortex and hippocampal CA1 area were detected in Rats after 3, 6, and 9 weeks of CCH. RES activates autophagy, probably through down-regulation of the PI3K/AKT/mechanistic target of the rapamycin (mTOR) pathway. RES inhibited neuronal apoptosis detected by TUNEL staining and protein expression of Bcl-2, Bax, and cleaved-caspase3. Reduced levels of oxidative stress factors MDA, SOD, and GSH were observed in RES administered CCH group (Wang et al., 2019). It is shown in these previous studies that multiple molecular mechanisms are involved in the neuroprotection afforded by RES after CCH, namely activating autophagy, anti-oxidation, anti-apoptosis, and reversing the synaptic plasticity deficits; however, other pathways might also take part in it. Despite differences in drug-dose, drug-administration approach, deliver duration, and observation time points, our observations coincide with previous findings that RES restored cognitive deficits and reduced hippocampal neuronal cell death. We further extend the possible mechanism of RES on neuroinflammation in CCH rats. We reported here that RES promotes the myelin integrity, reduced excess expression of reactive astrocytes and activated microglia, and inhibited the levels of myeloperoxidase and neuroinflammatory mediators, possibly through mitigation of the STING/TBK1/IRF3 pathway.

This study has several limitations. First, we find that hypoperfusion-induced ER stress is responsible for the activation of the STING pathway, but the exact molecules or mechanism of action that might be involved in STING activation in the 2VO model warrant further investigation. Given the various biological effects of STING, it will be important to find the optimal therapeutic levels to activate the STING pathway in a beneficial way. Elevation of STING axis expression was observed in neurons, microglia, and astrocytes, while their interplay is not clear. Further studies regarding the effects of STING/TBK1/IRF3 axis on CCH should be performed with a larger number of animals and other higher-order species. Second, although ER stress is proved in our CCH model, the cells which are primarily responsible for inducing ER-stress remain elusive. And the interaction between ER stress and the STING/TBK1/IRF3 axis warrants further study. Third, given the variety of subtypes and morphologies, the role of astrocytes and microglia in VaD warrants a more detailed investigation. Last, based on the above findings, the neuroprotective efficacy of RES was at least partly linked to STING/TBK1/IRF3 signaling inhibition, but some studies indicate that RES might target TBK1 to exert an anti-inflammatory effect. A previous study showed that RES enhances anti-inflammatory activity, decreases inflammatory cytokine levels by affecting microglial and astrocyte signaling pathways, and increases oligodendrocyte survival in stroke models. Based on well-documented evidence, RES exerts potential protective effects against extended and unrestricted ER stress (Ahmadi et al., 2021). Thus, RES may affect multiple targets, and the precise target and mechanism of RES during CCH merits further exploration.

In summary, CCH induces a robust neuroinflammatory response that is associated with increased expression of the STING cascade. RES reduces neuroinflammation, resulting in cognitive function recovery. STING pathway might participate in the neuroprotective effect of RES in CCH of Rats. These studies suggest that therapeutic modulation of the STING/TBK1/IRF3 pathway may limit persistent neuroinflammation and the development of cognitive impairment following CCH.

## Data availability statement

The datasets presented in this study can be found in online repositories. The names of the repository/repositories and accession number(s) can be found in the article/Supplementary Material.

## Ethics statement

The animal study was reviewed and approved by Animal Ethical Committee of Hebei General Hospital.

## Author contributions

PL, NK, and WJ contributed to conception and design of the study. NK, JS, YS, FG, YG, and MF performed the experiments. NK and FG performed the statistical analysis. NK wrote the manuscript. PL revised the manuscript. All authors contributed to manuscript revision, read, and approved the submitted version.

## Conflict of interest

The authors declare that the research was conducted in the absence of any commercial or financial relationships that could be construed as a potential conflict of interest.

## Publisher’s note

All claims expressed in this article are solely those of the authors and do not necessarily represent those of their affiliated organizations, or those of the publisher, the editors and the reviewers. Any product that may be evaluated in this article, or claim that may be made by its manufacturer, is not guaranteed or endorsed by the publisher.
