# Supplementary material for: Resveratrol reduces inflammatory response and detrimental effects in chronic cerebral hypoperfusion by down-regulating stimulator of interferon genes/TANK-binding kinase 1/interferon regulatory factor 3 signaling
Source: Front Aging Neurosci. 2022 Jul 22;14:868484. doi: 10.3389/fnagi.2022.868484 (PMC9354401; doi:10.3389/fnagi.2022.868484)
Supplement: Supplementary file 1 [file Data_Sheet_1.docx]

**Supplemental Figures**

1.To verify the specificity of the STING pathway, we performed 2VO surgery and administered rats with the STING-specific inhibitor H-151. Phosphorylated and total IRF3, TBK1 and STING were assessed by western blotting.

**
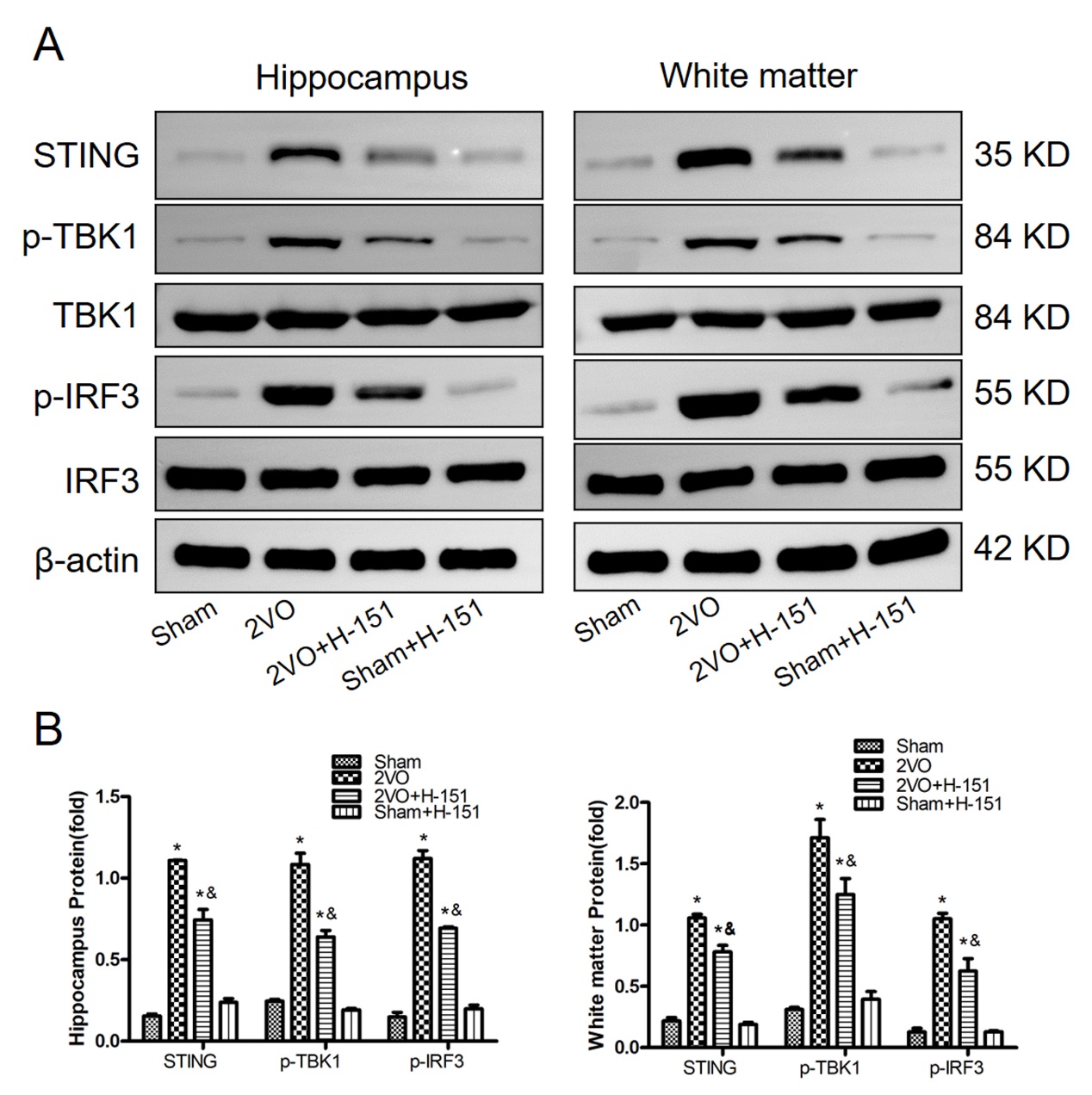
**

Supplementary Figure S1. Representative images (A) and quantitative analysis (B) of protein levels of STING, TBK1, IRF3, p-TBK1 and p-IRF3 in hippocampus and white matter extract at 4 weeks after 2VO. β-actin was used as an internal control (*n* = 3 per group). **p* < 0.05 versus Sham. &*p* < 0.05, 2VO+H-151 group versus 2VO group. Data are presented as means ± SD.

2. The levels of 2′3′-cGAMP, which is naturally synthesized by activated cGAS, were quantified by ELISA kit.


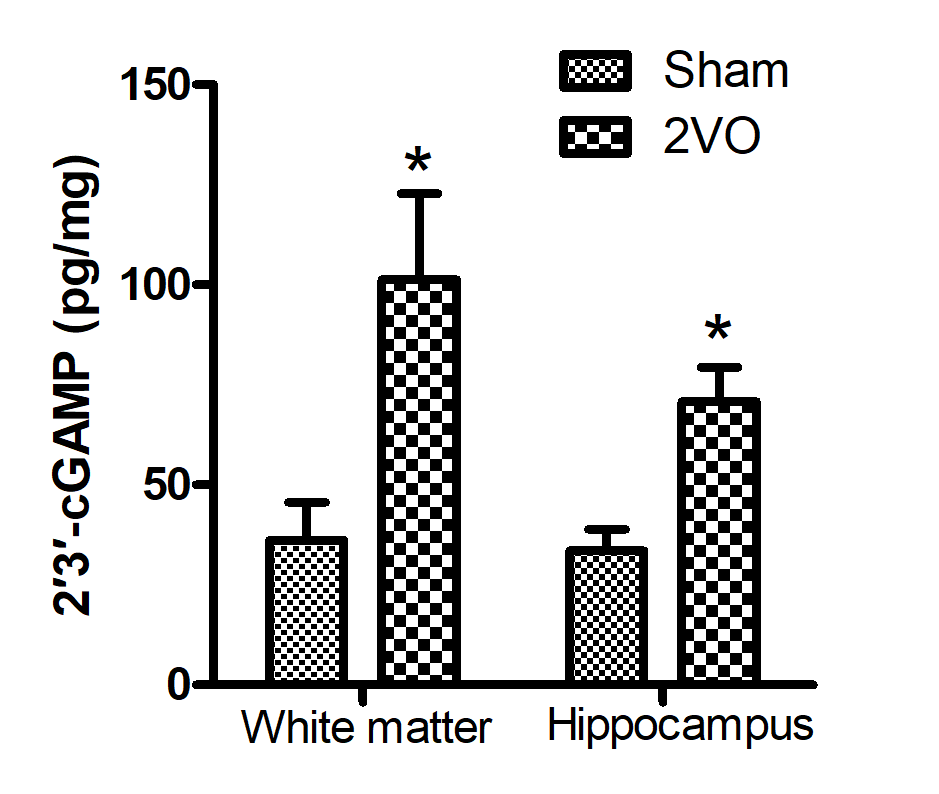


Supplementary Figure S2. ELISA of 2′3′-cGAMP levels in brain tissue homogenates of rats in diverse groups. The 2′3′-cGAMP level was quantified as pg/mg protein. Data are presented as mean ± SD (*n* = 3). * *p* < 0.05 stood for statistical significance.
